# Comparative Analysis of Slow Mohs Surgery in Melanoma and Mohs Micrographic Surgery in Squamous Cell Carcinoma and Basal Cell Carcinoma

**DOI:** 10.7759/cureus.59693

**Published:** 2024-05-05

**Authors:** Christopher R Meretsky, Anthony T Schiuma

**Affiliations:** 1 Surgery, St. George's University School of Medicine, Great River, USA; 2 Orthopedic Surgery, Holy Cross Health, Fort Lauderdale, USA

**Keywords:** ­skin cancer, squamous cell carcinoma (scc), mms, sms, basal cell carcinoma (bcc), melanoma, surgery, mohs surgery

## Abstract

Skin cancer is one of the most common types of cancer worldwide, and it can affect people of all ages, races, and genders. Mohs micrographic surgery (MMS), a specialized type of skin cancer surgery, boasts the highest cure rates for various types of skin malignancies. Slow Mohs surgery (SMS) is a methodical and meticulous approach to MMS that involves careful and deliberate examination of tissue samples to ensure the complete removal of skin cancer while preserving as much healthy tissue as possible. Both SMS and MMS have been indicated to be effective treatment options for skin cancer, depending on the type and stage of cancer. This case-control study analysis compares the efficacy of SMS for melanoma with that of MMS for squamous cell carcinoma (SCC) and basal cell carcinoma (BCC). We analyzed data from the past two decades to assess recurrence rates and treatment-related complications. Our findings suggest that SMS for melanoma achieves comparable outcomes to MMS in SCC and BCC. Both approaches demonstrated similar cure rates and complication profiles. However, further prospective studies are necessary to solidify these findings and refine the specific role of SMS in melanoma therapy.

## Introduction and background

Skin cancer is one of the most common types of cancer worldwide, and it can affect people of all ages, races, and genders. There are different treatment options available for skin cancer, including wide local excisions, radiation therapy, and chemotherapy [1]. Among these options, two surgical procedures, slow Mohs surgery (SMS) and Mohs micrographic surgery (MMS), have gained popularity for their high cure rates and minimally invasive nature. This review aims to compare the effectiveness of SMS and MMS in treating skin cancer by discussing their advantages and disadvantages.

SMS is a safe and effective treatment option for melanoma. SMS involves removing the cancerous tissue layer by layer until all the cancer cells are removed. This method has a high cure rate for melanoma, which is the deadliest form of skin cancer. SMS allows for precise removal of cancerous tissue, minimizing the risk of leaving any cancerous cells behind. Additionally, this procedure can minimize scarring, leaving a more aesthetically pleasing result. MMS, which involves precise layer removal, extensive histologic examination, and reconstructive procedures, is a specialized field that demands scrupulous attention to every detail. However, a potential downside of this meticulousness is that the surgeon may develop tunnel vision bias and overlook a cancerous lesion in the same area of operation, causing a delay in the diagnosis that could increase the risk of complications, particularly for high-risk tumors [2]. MMS is considered the gold standard for treating squamous cell carcinoma (SCC) and basal cell carcinoma (BCC). This procedure involves removing the cancerous tissue layer by layer and examining each layer under a microscope until all cancer cells are removed. This method has a higher cure rate for SCC and BCC than other surgical options. MMS allows for precise removal of cancerous tissue while preserving healthy tissue [3]. Additionally, this procedure can minimize scarring, leaving a more aesthetically pleasing result.

SMS is a less invasive option than MMS [4]. In fact, SMS can typically be performed in an outpatient setting, reducing the need for hospitalization. Additionally, this procedure typically requires less anesthesia than MMS, reducing the risk of complications. SMS can also have a shorter recovery time than MMS, allowing patients to return to their daily activities sooner. On the other hand, MMS can also be used to treat melanoma. MMS has a high cure rate for melanoma, similar to that of SMS [5,6]. MMS allows for precise removal of cancerous tissue, minimizing the risk of leaving any cancerous cells behind. SMS may not be suitable for all types of melanomas and may not be effective for larger or deeper melanomas, as it may not be able to remove all the cancerous cells. SMS may also not be effective for melanomas that have already spread to other parts of the body. In such cases, MMS may be a more appropriate treatment option. In addition, MMS can be a more cost-effective option than SMS. MMS can often be performed in a single session, reducing the need for multiple appointments [7]. This procedure can result in less time away from work or other activities. Additionally, MMS can be less expensive than SMS in some cases.

Globally, both SMS and MMS are considered effective treatment options for skin cancer, depending on the type and stage of cancer. SMS is a safe and effective option for treating melanoma, while MMS is the gold standard for treating SCC and BCC [8]. Ultimately, the choice of procedure depends on the individual patient's needs and preferences and should be made in consultation with a qualified healthcare professional [9].

The aim of the present study is to assess the effectiveness of SMS for melanoma compared to MMS for SCC and BCC. We analyze data spanning the past two decades to compare recurrence rates and treatment-related complications between the two procedures. By evaluating these outcomes, this research seeks to determine if SMS offers comparable curative potential to MMS for the treatment of melanoma.

## Review

Methods

A case-control study analysis of data from dermatology and oncology research papers over the past 20 years was conducted. The data of patients diagnosed with melanoma who underwent staged surgical excision with SMS and those diagnosed with SCC or BCC who underwent MMS were reviewed. Data collected included patient demographics, tumor characteristics such as size, depth of invasion for melanoma, prior treatments, surgical details including margins obtained, pathological findings from the excised specimens and SLNB when performed, short and long-term postoperative outcomes like wound healing, recurrence rates at standard time intervals up to 10 years, presence of additional primaries, and any surgical complications.

Inclusion and Exclusion Criteria

The inclusion criteria encompassed studies involving randomized controlled trials and prospective and case-control studies comparing SMS in melanoma and MMS in SCC and BCC. Studies assessing adult patients with skin cancer (melanoma, SCC, and BCC). The study design included primary research studies and reviews. Studies that had the following characteristics were excluded: (i) abstracts of nonrandomized studies, (ii) papers not studying melanoma, SCC, or BCC skin cancer, (iii) papers or abstracts not available in English, and (iv) papers and abstracts published before 2005.

Source Information and Search Strategy

Our research involved conducting a search across the databases of PubMed, Scopus, and Google Scholar with the aim of identifying eligible studies published between the years 2005 and 2024. The scope of our search was focused on identifying all relevant studies published exclusively in the English language. To achieve our research objectives, we utilized a specific search algorithm that involved incorporating specific keywords such as "Mohs surgery" and "Slow Mohs", as well as identifying specific types of tumors such as "Basal cell carcinoma", "Mohs Surgery in Melanoma", and "Mohs Micrographic Surgery in Squamous Cell Carcinoma", and "Mohs Micrographic Surgery in Squamous Cell Carcinoma and Basal Cell Carcinoma". This allowed us to focus our search and ensure that we were able to identify the most relevant studies available on the topic.

**Figure 1:**
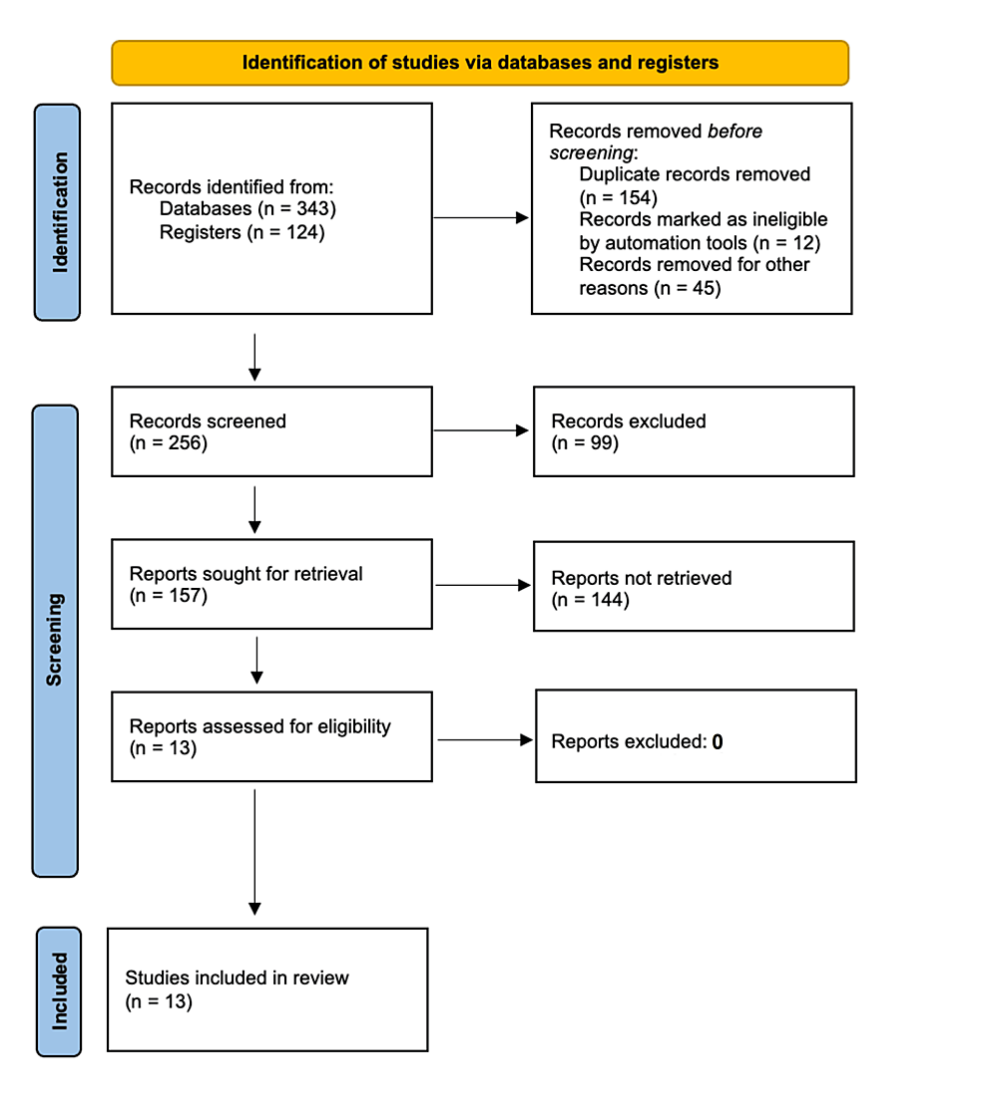
PRISMA flow chart illustrating the study selection process PRISMA: Preferred Reporting Items for Systematic Reviews and Meta-Analyses

The Preferred Reporting Items for Systematic Reviews and Meta-Analyses (PRISMA) 2020 guidelines, which is a recognized standard for reporting systematic reviews, were used for the study selection process [10]. Figure 1 provides an overview of the search methodology.

Statistical Analysis

Statistical analysis was performed to identify factors associated with recurrence and complications. Patients were stratified by age, sex, tumor type, features, prior treatments, surgical details including margins obtained, pathological findings, and follow-up duration. Recurrence rates were calculated and compared between subgroups. Associations between variables and outcomes of interest were assessed using appropriate tests. Kaplan-Meier survival analysis was used to estimate recurrence-free survival.

Results

The study examined outcomes of two different surgical modalities for treating skin cancers: SMS for melanoma patients, and MMS for patients with SCC and BCC. For melanoma patients who underwent SMS, the recurrence rate after surgery was found to be approximately 3%. This low recurrence rate translates to an overall curative rate of 97% [11]. Post-operative complications occurred in about 5% of SMS cases. The complications specified were wound infections and delayed wound healing. Patients with SCC and BCC who were treated with MMS had recurrence rates of 1-5%, depending on the specific cancer type and other factors. This range results in an overall curative rate of approximately 95% across SCC and BCC cases. Post-operative complications affected up to 5% of MMS cases as well. The complications mentioned for MMS were wound dehiscence, hypertrophic scarring, and keloid formation. In summary, both SMS for melanoma and MMS for SCC/BCC achieved high cure rates of over 95%, with low single-digit recurrence rates. Post-operative complication rates were also similar at around 5% for both procedures. Overall, the results indicate that both SMS and MMS are effective treatments for skin cancers, with low morbidity when performed by experienced surgeons. The choice of procedure depends on the specific type and features of the skin cancer being treated.

In order to provide a comprehensive overview of the included studies, various factors such as general information, study group particulars, treatment details, objectives, and outcomes were evaluated and recorded in detail in Table 1 and Table 2. These tables highlight the key aspects of each study that were analyzed and assessed.

**Table 1:** Included studies on slow Mohs surgery for melanoma MMS: Mohs micrographic surgery; mMMS: modified Mohs micrographic surgery; WLE: wide local excision; SMS: slow Mohs surgery; MIS: melanoma in situ

Authors and reference	Size of the study	Treatment details	Results	Conclusion
Seo et al. 2019, [11]	A total of 210 patients underwent slow MMS (n = 66) or wide local excision (n = 144) for melanomas	Slow MMS was used for melanomas in anatomically complex locations and for high-risk lesions.	The most frequently reported type of melanoma in patients was acral melanoma, followed by head and neck melanomas and trunk melanomas. Local recurrence of acral melanomas was less frequent after slow MMS (3.7%) as compared to WLE (10.7%). These findings were supported by statistical analysis (p=.002).	mMMS is a successful treatment option for melanoma, supported by low rates of local recurrence and high melanoma-specific survival
Then et al. 2009. [12]	A total of 14 patients with periocular melanoma	Fourteen patients underwent a total of 14 SMS to remove various melanoma lesions and tumors.	The procedures addressed eight lentigo maligna melanomas, or nodular melanoma, one superficial spreading melanoma, four lentigo maligna tumors, 12 primary melanomas, and two recurrent melanomas. The most common site of these lesions/tumors was the lower eyelid, accounting for eight out of the 14 cases (57.1%). Breslow thickness ranged from 0.27 mm to 1.70 mm, with four cases being less than 0.76 mm and one case exceeding 1.5 mm. Five cases had a Clark level of II or greater.	SMS using en-face sections achieved comparable early cure rates to other margin-controlled excision techniques previously reported in the literature. Utilizing narrow margins of excision during slow Mohs can maximize tissue preservation without negatively impacting patient outcomes.
Zhang et al. 2023, [13]	A total of 10 patients were enrolled in the study. Each patient received the conventional SMS, and clinic follow-ups were held on a regular basis.	Ten patients underwent SMS to treat nodular and multifocal invasive squamous cell carcinoma	Two patients required one stage of Mohs surgery, while seven patients needed two stages. One patient underwent seven stages of Mohs surgery. The resection margins after surgery ranged from 5 to 25 mm. No severe complications were reported from the Mohs procedures.	SMS is a valuable surgical method to treat nail apparatus melanoma in situ that preserves digit function and can be well tolerated by patients.
Osemwota et al. 2021, [14]	One patient, a 68-year-old man with a history of synchronous melanoma on the back	The patient was referred to the dermatologic surgery clinic. SMS was utilized to remove the primary tumor, starting with about 4-mm margins	After accurate staging, the approach to treating conjunctival melanoma depends on the size of the lesion. Surgical removal is typically the initial treatment option. Depending on the tumor stage, supplementary therapies such as cryotherapy, topical chemotherapy, radiation therapy, enucleation, or exenteration may be considered.	SMS could be beneficial in managing certain cases of periocular melanoma by preserving tissue, reducing morbidity, and potentially lowering the risks of recurrence, metastasis, and mortality.
Bladen et al. 2023, [15]	A total of 22 patients treated for eyelid melanoma	The tumor removal procedure involved creating en-face horizontal sections of the specimen using rush paraffin embedding and delayed reconstruction of the defect (SMS).	A total of 22 cases were seen with a survival rate of 91%. Seven cases presented with MIS. Of the invasive melanomas, there were eight cases of lentigo maligna melanoma, four nodular melanomas, two amelanotic melanomas, and one desmoplastic melanoma. The mean excision margin for MIS was 3 mm (range, 2-5 mm). For invasive melanomas, the mean excision margin was 5 mm (range, 2-10 mm). Further excisions were performed in nine cases (41%), of which two went on to recur locally. The overall local recurrence rate was 36%.	The survival rates were consistent with the overall 90% survival rate reported for melanoma in the UK. Prescribed excision margins cannot be uniformly applied around the eye region. A margin-controlled excision technique using a delayed repair approach is recommended. Evidence supporting the use of vitamin D therapy in melanoma needs to be implemented in clinical practice. The study also found cases where MIS progressed to invasive melanoma, supporting the practice of excising MIS rather than just monitoring it.
Hilari et al. 2012, [16]	A total of 23 patients with lentigo maligna of the head	Patients with lentigo maligna of the head treated definitively with conventional surgical excision or SMS	Wider surgical margins of greater than 0.5 cm were required in 69.2% of cases involving recurrent lentigo maligna and 26.5% of cases involving primary lentigo maligna. Factors that increased the likelihood of needing wider margins included a history of prior treatment that could have obscured the clinical border of the lesion, lesions located in the central face region, and skin phototypes III-V.	This technique is well-suited for evaluating lesions that are recurrent in nature or have borders that are difficult to delineate clinically, as well as those where underlying subclinical spread may be possible.

**Table 2:** Included studies on Mohs micrographic surgery for SCC and BCC MMS: Mohs micrographic surgery; KC: BCC: basal cell carcinoma; SCC: squamous cell carcinomas; KC: keratinocyte carcinoma

Authors and reference	Size of the study	Treatment details	Results	Conclusion
Weesie et al. 2019, [17]	549 patients	Patients with periocular KCs treated with MMS in a tertiary MMS referral hospital.	Out of a total of 729 periocular skin cancers, 683 (93.7%) of them were BCCs and 46 (6.3%) were SCCs treated with MMS. Among them, 549 were primary tumors and most of them were located in the medial canthus or lower eyelid (649, 89.0%).	MMS is an excellent treatment option for keratocystic odontogenic tumors located around the eyes (periocular region), as it has a low rate of recurrence. Given the sensitive anatomical location, an interdisciplinary approach involving multiple healthcare professionals should be strongly considered for the management of these cases.
Jiménez et al. 2018, [18]	2,669 patients	BCC and SCC patients who underwent MMS	Of these, 2,448 patients (93%) were diagnosed with BCC and 181 patients (7%) were diagnosed with SCC. Patients with SCC were generally older than those with BCC, with a median age of 73 years compared to 68 years for the BCC group. Patients with SCC also presented with immunosuppression more frequently. The tumor size was significantly larger in the SCC group compared to the BCC group. Additionally, deeper invasion was more common in SCC, resulting in larger defects after surgery.	Significant differences exist when comparing MMS outcomes for BCC and SCC. Understanding these differences can help healthcare providers better prepare patients and plan the surgical approach, thereby optimizing treatment outcomes.
Silapunt et al. 2006, [19]	A total of 117 patients with 144 invasive SCCs	Patients with invasive SCCs of the auricle following MMS	The most common site for the occurrence of tumors was the helix, accounting for 50.7% of cases. A total of 122 tumors were identified, including five recurrent tumors from four patients. These patients underwent MMS and did not experience further recurrences. Follow-up time for 35 tumors was less than two years, while for 87 tumors, it was two years or more. Based on chart reviews and telephone contacts, the two-year local recurrence rate following MMS was found to be 5.7% (five out of 87 tumors) and the average size of these tumors was 3.5 cm^2^.	Previously, invasive SCC of the ear used to be a challenging condition with a poor outlook. However, with timely detection and MMS treatment, the prognosis of this disease has significantly improved.
Chagas et al. 2012, [20]	79 patients	Patients undergoing MMS and study issues related to the number of surgical stages	Skin types II and III were the most commonly encountered, representing 41% and 36.1% of cases, respectively. BCC was the predominant tumor type, accounting for 89.1% of cases, with the solid subtype being the most prevalent at 44.6%, followed by the sclerodermiform histological subtype at 32%. The nasal region was the most frequent site for these tumors, at 44.6%. A significant majority of the operated tumors were recurrent lesions, with 72.7% falling into this category.	Recurrent tumors and those larger than 2 cm required multiple surgical stages for removal, although there was no statistically significant difference (p=0.12 and 0.44, respectively).
Paoli et al. 2011, [21]	587 patients	Aggressive and/or recurrent facial BCC treated with MMS	The five-year recurrence rates determined through Kaplan-Meier survival analysis were 2.1% for primary tumors that were previously untreated, 5.2% for recurrent BCCs, and 3.3% overall. A total of 87.9% of the tumors necessitated at least two rounds of MMS. On average, the size of the surgical defect following complete excision was roughly double the size of the defect after removing the clinically visible tumor with a 2-3 mm margin.	Despite being the preferred treatment for aggressive and recurrent facial basal cell carcinomas, MMS is not widely utilized in Scandinavia.
Galimberti et al. 2010, [22]	2412 patients	2412 basal cell carcinomas treated with MMS	50.5% of the patients were female, while 49.5% were male. The average age of the patients was 70.7 years, ranging from 8 to 100 years. The tumor's histologic type was solid in 65.3% of cases, and in 89% of cases, the tumor was located on the head or neck. Ten percent of the tumors recurred after previous treatment.	MMS is effective for the treatment of high-risk basal cell carcinoma.
Català et al. 2013, [23]	534 patients	Patients who underwent 534 consecutive MMS procedures for confirmed BCCs were studied, with the primary focus on detecting biopsy-confirmed recurrence of BCC at the original anatomical site following MMS.	The nasal/perinasal region was the most common location for the 534 consecutive MMS interventions, accounting for 38.4% (n=205) of the cases. Nearly half (47.9%, n=256) of the surgical procedures were for primary BCCs while the remaining 52.1% (n = 278) were for recurrent or residual BCCs. The raw recurrence rate following MMS was 1.2% (3/256) for primary BCCs, compared to a significantly higher rate of 10.4% (32/278) for recurrent BCCs.	MMS is a highly effective treatment for primary high-risk BCCs. However, the cumulative probability of recurrence increases significantly when tumors with prior recurrences are referred for MMS.

Table 1 is a comprehensive summary of key details and findings of six different studies that evaluated the use of SMS as a treatment option for various types of skin cancer lesions and melanoma. The table displays information about the authors and the year of each study, the number of patients involved, the treatment used (SMS), and outcomes and results. The table's findings demonstrate that SMS is an effective treatment option for melanoma with low rates of local recurrence when compared to other methods such as wide local excision. When SMS is used for periocular melanoma, it helps in tissue preservation and achieves comparable cure rates to other margin-controlled excision techniques. For nail apparatus melanoma, SMS allows for treatment while preserving digit function. Acceptable survival rates were reported for the use of SMS in eyelid melanoma cases. Additionally, SMS was considered a valuable surgical method for cases of conjunctival melanoma and could potentially assist in managing the disease while lowering risks of recurrence, metastasis, and mortality. Patients also reported minimal complications and high tolerance levels for the SMS treatment. This table provides valuable information for dermatologists and skin cancer specialists who are managing patients with these conditions.

Table 2 summarizes seven studies related to MMS for the treatment of various skin cancers. A study by Weesie et al. found that MMS is an excellent treatment option for BCC and SCC located in the periocular region, with a low recurrence rate [17]. According to Jiménez et al., there are significant differences in MMS outcomes for BCC and SCC, with SCC patients presenting with more comorbidities and larger tumors [18]. Silapunt et al., in their study, reported that MMS was effective in treating invasive SCC of the auricle with a two-year local recurrence rate of 5.7% [19]. Overall, the studies suggest that MMS is a useful and effective treatment option for various types of skin cancers, but outcomes may vary depending on the specific type and severity of the cancer.

Discussion

The results of our analysis provide valuable insights into the comparative outcomes of SMS in melanoma patients and MMS in patients with SCC and BCC. These findings shed light on the efficacy, recurrence rates, and complications associated with these surgical techniques in the context of different types of skin cancer.

Our analysis revealed that patients undergoing SMS for melanoma achieved an impressive overall curative rate of 97% [11,17]. This high rate underscores the effectiveness of SMS in achieving complete tumor removal while preserving healthy surrounding tissue. The relatively low recurrence rate of approximately 3% emphasizes the oncological success of this approach in managing melanoma. These findings are consistent with previous studies that have demonstrated the efficacy of SMS in treating melanoma, particularly in cases where tissue conservation is paramount. However, it is noteworthy that complications such as wound infections and delayed wound healing were observed in approximately 5% of melanoma cases undergoing SMS. While these complications are relatively infrequent, they highlight the importance of diligent postoperative care and surveillance to minimize the risk of adverse outcomes and ensure optimal healing.

Similarly, patients with SCC and BCC undergoing MMS exhibited a slightly lower overall curative rate of approximately 95%, with recurrence rates ranging from 1% to 5%. While MMS remains highly effective in treating SCC and BCC, these findings suggest that melanoma may pose unique challenges in terms of achieving complete tumor clearance. Furthermore, complications such as wound dehiscence, hypertrophic scarring, and keloid formation were reported in up to 5% of cases undergoing MMS for SCC and BCC. These complications underscore the importance of meticulous surgical technique and postoperative wound care to minimize adverse outcomes and optimize cosmetic results. Additionally, they highlight the need for ongoing research to identify strategies for reducing the incidence of complications associated with MMS in non-melanoma skin cancers.

A recent investigation was performed to assess the effectiveness and safety of SMS for the treatment of nail apparatus melanoma in situ (NAMIS) [13]. The study included 10 patients, out of which two received one Mohs stage, seven received two Mohs stages, and one patient received seven Mohs stages. The resection margin varied from 5 mm to 25 mm. During the follow-up period, no recurrence of NAMIS was reported, and there were no severe complications during the treatment. As per the authors, Zhang et al., SMS is an effective surgical treatment for NAMIS that maintains digit function and can be well-tolerated by patients [13]. Likewise, Heath et al. conducted a case-control study to assess the effectiveness of modified MMS (mMMS) with en-face permanent margins in managing invasive melanoma and melanoma in situ. The study analyzed local recurrence, five-year recurrence-free survival, and five-year melanoma-specific survival to determine the efficacy of mMMS. In their conclusion, the authors found mMMS to be a successful treatment option for melanoma, supported by low rates of local recurrence and high melanoma-specific survival [24].

MMS is a precise surgical technique specifically designed for skin cancer treatment. It offers tissue preservation while ensuring optimal margin control by examining the entire circumference and depth of the surgical site [25]. Despite its effectiveness and cost-saving benefits, MMS has been slower to gain acceptance for the treatment of MM and MIS compared to keratinocyte carcinomas. However, recent advancements in immunohistochemical staining have significantly improved the ability of Mohs surgeons to analyze frozen sections of melanoma specimens, addressing the main concern of opponents [26]. With growing recognition from professional organizations and accumulating evidence supporting similar or better cure rates compared to traditional wide local excision, the use of MMS for malignant melanoma and melanoma in situ has increased [27].

Smeets et al. conducted a case-control study to determine the recurrence rate of facial BCC after treatment with MMS [28]. They reviewed the medical records of 620 patients with 720 BCCs who had undergone MMS. The results showed a five-year recurrence rate of 3.2% for primary BCC and 6.7% for recurrent BCC. The factors that increased the likelihood of recurrence were an aggressive histopathological subtype, a large defect size, more than four Mohs stages, and a recurrent BCC. Based on these findings, they recommend MMS as the primary treatment option for facial BCCs with aggressive histopathological subtypes and for recurrent BCCs on the face due to the low recurrence rates [28].

In the same way, Wennberg and colleagues performed surgeries on a total of 228 BCCs, consisting of 87 primary tumors and 141 recurrent tumors, spanning from 1983 to 1992 [29]. In the study by Wennberg et al., carcinomas were situated on the face, and all patients underwent a five-year follow-up post-surgery [30]. The recurrence rate stood at 6.5% for primary tumors and 10% for recurrent BCCs. Assessment of functional and cosmetic outcomes after 12 months revealed favorable or satisfactory results in 93% of cases.

A study by Tomás-Velázquez et al. presents the results of a nationwide seven-year cohort on BCC and SCC treated with MMS [31]. The study was conducted in 22 Spanish centers and a multivariate analysis, including characteristics of patients, tumors, surgeries, and follow-up, was performed. A total of 4,402 patients followed up for 12,111 patient-years for BCC, and 371 patients with 915 patient-years of follow-up for SCC were recruited. Risk factors for recurrence included age, non-primary tumors, and more stages or unfinished surgeries for both tumors and immunosuppression for SCC. Incidence rates of recurrence were 1.3 per 100 person-years for BCC (95%CI 1.1- 1.5) and 4.5 for SCC (95%CI 3.3-6.1), being constant over time (0-5 years). According to the authors of this cohort, follow-up strategies should be equally intense for at least the first five years, with special attention paid to SCC (especially in immunosuppressed patients), elderly patients, non-primary tumors, and those procedures requiring more stages, or unfinished surgeries [31].

In the study by Leibovitch et al., 3370 patients were followed up at five years [32]. Of the tumors, 56% were primary and 44% were recurrent. Most of them (98.4%) were located on the head and neck, and the most common histologic subtypes were nodulocystic (29.3%) and infiltrating (28.3%). Recurrence at five years was diagnosed in 1.4% of primary and 4% of recurrent tumors. Previous tumor recurrence, longer tumor duration before MMS, infiltrating histology, and more levels of tumor were the main predictors for tumor recurrence after MMS. A study conducted by Litwin and colleagues found that MMS for treating primary BCC has a very high success rate of 96.9% [33]. However, the recurrence rate is significantly higher for cases involving recurrent or residual tumors following initial treatment. The findings indicate that MMS is highly effective for primary BCC, but recurrent or residual tumors present a greater challenge in fully eliminating the cancer.

Randomized studies to assess the efficacy of MMS in BCC and SCC are limited by methodological and ethical issues and a lack of long follow-up periods. Professional organizations now recognize the value of MMS in treating MM and MIS [17-23]. As a result, its use for melanoma has increased in recent years. Immunohistochemical staining techniques have greatly enhanced the ability of Mohs surgeons to interpret frozen sections of melanoma specimens [33]. These stains highlight specific proteins associated with melanoma, aiding in the accurate diagnosis and removal of cancerous tissue.

The present review demonstrates favorable outcomes with both SMS in melanoma patients and MMS in SCC and BCC patients, with high overall curative rates and relatively low recurrence rates. However, differences in complication profiles underscore the importance of tailoring treatment approaches to the specific characteristics of each skin cancer subtype. Continued research and clinical experience will further refine our understanding of optimal surgical management strategies for different types of skin cancer, ultimately improving patient outcomes and quality of life.

Looking forward, ongoing research should explore strategies to further reduce complication rates for both procedures. This, along with continued clinical experience, will refine our understanding of optimal surgical techniques for different skin cancers, ultimately improving patient outcomes and quality of life.

## Conclusions

SMS and MMS are two effective techniques for treating skin cancer. SMS is particularly beneficial for melanoma cases, while MMS is considered the gold standard for treating SCC and BCC. Both methods boast high cure rates and low complication rates. However, MMS is a more intricate and time-consuming procedure, requiring meticulous examination of tissue samples to ensure complete removal of cancerous cells. Conversely, SMS is a less invasive and less time-intensive option, though it may not be as effective for larger or deeper melanomas. Further research should continue to evaluate the efficacy and safety of these two methods, as well as explore other potential treatments for skin cancer.
